# Comparison of Virulence Patterns Between *Streptococcus uberis* Causing Transient and Persistent Intramammary Infection

**DOI:** 10.3389/fvets.2022.806674

**Published:** 2022-04-18

**Authors:** Anyaphat Srithanasuwan, Noppason Pangprasit, Witaya Suriyasathaporn

**Affiliations:** ^1^Department of Food Animal Clinic, Faculty of Veterinary Medicine, Chiang Mai University, Chiang Mai, Thailand; ^2^Akkhraratchakumari Veterinary College, Walailak University, Nakhon Si Thammarat, Thailand; ^3^Research Center of Producing and Development of Products and Innovations for Animal Health and Production, Chiang Mai University, Chiang Mai, Thailand

**Keywords:** virulence factor, persistent intramammary infection, transient intramammary infection, *Streptococcus uberis*, mastitis

## Abstract

The objectives of this study were determined by two experiments including Experiment 1 (EXP1) using *Streptococcus uberis* obtained from a weekly longitudinal study to compare virulence patterns between transient and persistent intramammary infection (IMI), and Experiment 2 (EXP2) using a stored-known-appearance PFGE strain of a contagious *S. uberis* to determine a change of virulence patterns after long-term transmission. For EXP1, quarter milk samples from 31 milking cows were aseptically and longitudinally collected once a week for 10 weeks. A total of 14 *S. uberis* isolates from quarters with 1 and >4 weeks of duration of IMI were categorized as transient and persistent IMI, respectively. For EXP2, 11 isolates of a stored-known-appearance PFGE strain of *S. uberis* from our previous study (1) were randomly selected, including 5 from transient IMI (1 month) and 6 from persistent IMI (>1 month). The virulence profiles of all isolates were investigated, including *sua, hasAB, hasC, gapC, pauA*, and CAMP factor or *cfu*, using PCR. The Kaplan–Meier estimates were used to calculate the duration of IMI in EXP1. Approximately 50% of field *S. uberis* IMI was spontaneously cured within 1 week, while 25% was not cured within 10 weeks. From EXP1, 4 virulence patterns were found in 14 isolates. The majority of patterns for transient *S. uberis* did not include *hasAB* (63.6%), the gene relating to capsule formation. Regardless of transient or persistent IMI, a high similarity of the virulence pattern within a PFGE strain was found in EXP2. Few changes of virulence pattern within a PFGE strain were found or were related to its subsequently changing to transient IMI.

## Introduction

Mastitis is a costly disease in the dairy industry. Several factors influence the development of mastitis, including bacterial virulence and quantity of invading microbes, udder defense efficiency, and environmental risk factors. Being a bacterial disease, mastitis can self or spontaneously cure as a result of the udder defense mechanisms (1). Based on the intramammary infection (IMI) duration before their spontaneous cures, mastitis appears as up to 1 and >1 month of IMI duration, the so-called transient and persistent IMI, respectively (2), in which a persistence longer than 2 months is termed chronic (1, 3). *Streptococcus uberis* is a common environmental mastitis pathogen, but many molecular studies indicate contagious transmission (1, 4). Contagious pathogens, such as *Streptococcus agalactiae*, more frequently appear as persistent IMI than other environmental mastitis-causing bacteria (5). In contrast, environmental *S. uberis* strains cause transient IMI, although contagious *S. uberis* strains may appear as transient IMI, persistent IMI, or both (1).

The great diversity of mastitis severity outcomes is thought to be due largely to the host (1, 3, 6). For *S. uberis* mastitis, some studies support the existence of specific persistent strains (2) whereas others do not, leading to the suggestion that cow factors rather than strains determine the duration of infection (2). Our previous study hypothesized both theories, as some *S. uberis* PFGE strains appeared separately, but some specific strains are both transient and persistent IMI, suggesting that the appearance was dependent on udder immune efficiency during IMI (1). Several putative virulence-associated genes of *S. uberis* causing mastitis were described (7), for example, resistance to phagocytosis conferred by a hyaluronic acid (HA) capsule (*hasAB*+*hasC*) (8), plasminogen activator proteins such as *PauA* (9), adherence and invasion to mammary epithelial cells mediated by SUAM (10), CAMP factor or *cfu* (7), and a surface dehydrogenase protein *gapC* (11). Several recent studies demonstrate the presence of virulence factors affecting pathogenicity and growth (12–16). However, the study on virulence factor patterns associated with transient or persistent *S. uberis* IMI has not been reported. In addition, for contagious *S. uberis*, changes in virulence factors in long-term transmission into other quarters or cows were hypothesized. Therefore, two studies were carried out including Experiment 1 (EXP1) using *S. uberis* obtained from a weekly longitudinal study to compare virulence patterns of 7 virulence factor genes, including *sua, hasAB, hasC, gapC, pauA*, and *cfu* of *S. uberis* between transient and persistent IMI, and Experiment 2 (EXP2) using a stored-known-appearance PFGE strain of a contagious *S. uberis* to determine a change of the virulence patterns after long-term transmission.

## Methods

### Sources of *Streptococcus uberis* Isolates

EXP1 was performed using a small dairy herd with high bulk somatic cell count (>1,000,000 cells/ml) and highly infected with *S. uberis* including 12 of 31 cows (38.7%) from 18 of 124 quarters as previously investigated by the Faculty of Veterinary Medicine, Chiang Mai University. After the farm selection, all quarter milk samples of all 31 milking cows were aseptically weekly longitudinally collected for bacterial identification for 10 weeks from September to November 2020. Bacterial identification was performed according to the National Mastitis Council (NMC) guideline. Samples with >3 species were excluded due to environmental contamination. Regardless of their clinical appearance, all quarters with *S. uberis* IMI were included in the study. An IMI episode was determined when *S. uberis* was first detected and continuously identified until its spontaneous cure. A quarter was considered to be spontaneously cured when continuous *S. uberis* samples were not presented in 14 days or 2 weeks later (17). The Kaplan–Meier estimates (18) were used to calculate the duration of *S. uberis* IMI. Time for a spontaneous cure began at the time *S. uberis* IMI was first detected and continued until *S. uberis* was cured spontaneously. Cases where only the starting point of infection was known, because the infection remained at the end of the study or it was ended early due to drying off, culling, or antibiotic treatment, were censored. The *S. uberis* isolates were randomly selected from quarters with 1 and >4 weeks of IMI duration, as transient and persistent episodes according to Pullinger et al. (2). The selected isolates were confirmed by matrix-assisted laser desorption/ionization time-of-flight mass spectrometry (MALDI-ToF MS) and stored at −80°C until use.

For EXP2, 11 stored-known-appearance PFGE strains of a contagious *S. uberis*, identified as PFGE type A as in the study of Leelahapongsathon et al. (1), were randomly selected and obtained from the −80°C stored bacteria container, including 5 isolates with 1-month IMI duration, and re-determined as transient episodes. Six isolates with >1 month of IMI duration were then determined as persistent episodes according to Pullinger et al. (2). In February 2021, all selected *S. uberis* isolates (*n* = 25) for both EXP1 and EXP2 were grown in Tryptic Soy Broth (TSB; HiMedia Laboratories Pvt. Ltd., Mumbai, India) and incubated overnight at 37°C. Genomic DNA was extracted using a Genomic DNA purification kit (Bio-Helix Co., Ltd., New Taipei City, Taiwan) and stored at −20°C until use.

### Molecular Analysis

All selected isolates confirmed *cpn60* gene (housekeeping gene) of *S. uberis* using conventional PCR (19). The seven primer pairs used for PCR amplification and the annealing temperatures are shown in Table 1. For PCR, the reaction mixture (25 μl) had a final concentration of 1× KOD OneTM PCR master Mix containing 1.25 U of Taq DNA polymerase (TOYOBO Co., Ltd., Osaka, Japan) and 0.4-μM concentrations of each forward and reverse primer, approximately 100 ng of genomic DNA, and DNase-free water. The PCRs to amplify target genes were as follows: initial denaturation at 95°C for 5 min, 30 cycles of denaturation at 98°C for 10 s and annealing for 5 s, extension at 68°C for 10 s, and final extension at 72°C for 10 min. The presence of PCR products was determined by electrophoresis of the reaction product in 1.5% agarose gel (Bio Basic Inc., Markham, ON, Canada) and using a 1.5-kb DNA ladder as a molecular marker.

**Table 1:** Primer sequences.

**Target genes**	**Primer function**	**Putative function**	**Primer sequence (5^′^-3^′^)**	**Annealing temperature (**°**C)**	**Product size (bp)**	**Ref**.
*cpn60*	Heat shock protein 60 (housekeeping gene)	Protein synthesis and metabolism	F-TCGCGGTATTGAAAAAGCAACAT R-TGCAATAATGAGAAGGGGACGAC	56	400	(19)
*sua*	Adhesion molecule	Adherence and invasion of mammary epithelial cells	F-ACGCAAGGTGCTCAAGAGTT R-TGAACAAGCGATTCGTCAAG	57	776	(7)
*hasA*	Hyaluronic acid capsule	Resistance to phagocytosis	F-GAAAGGTCTGATGCTGATG R-TCATCCCCTATGCTTACAG	55	319	(20)
*hasB*			F-TCTAGACGCCGATCAAGC R-TGAATTCCTATGCGTCGATC	56	532	(20)
*hasC*			F-TGCTTGGTGACGATTTGATG R-GTCCAATGATAGCAAGGTCAC	58	225	(20)
*gapC*	Surface dehydrogenase protein	Colonization	F-GCTCCTGGTGGAGATGATGT R-GTCACCAGTGTAAGCGTGGA	60	200	(7)
*pauA*	Plasminogen activator	Colonization	F-CGGGTTGAAGAACCTATCACTC R-TGGAAGTTGACCAAGAGAATTG	60	255	(21)
*cfu*	CAMP factor	Forms pores in host-cell membrane	F-TATCCCGATTTGCAGCCTAC R-CCTGGTCAACTTGTGCAACTG	50	205	(7)

## Results

**Figure 1:**
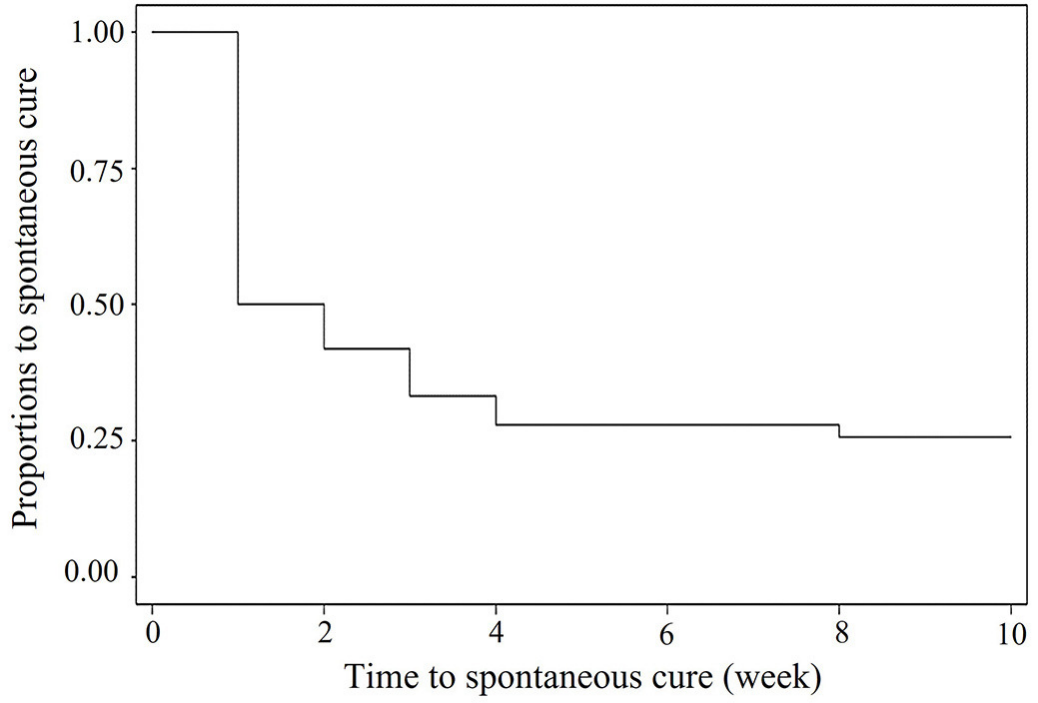
Kaplan—Meier survival curve of duration of field *Streptococcus uberis* IMI observed during a 10-weeks period.

For the 10-week field investigation in EXP1, 5 out of 31 cows were dried before the end of the study. Among the total of 1,141 milk samples, 221 samples from 74 quarters of 31 cows were infected with *S. uberis* including 90 new IMI ranging from 1 to 10 weeks with 25% of censor cases. A Kaplan–Meier curve on the duration of *S. uberis* IMI is shown in Figure 1. Approximately 50% of *S. uberis* IMI spontaneously cured within 1 week, while 25% was not spontaneously cured within 10 weeks. Fourteen *S. uberis* isolates were randomly selected including 1-week IMI as a transient episode (*n* = 11) and >4 weeks of IMI (n = 3) from 4, 6, and 9 consecutive weeks as persistent episodes for analysis of their virulence factor patterns. For EXP2, the 6 persistent episodes included two isolates from episodes with 2-month duration and each isolate from episodes with 3-, 5-, 6-, and 10-month duration.

From all 25 *S. uberis* isolates of both EXP1 (*n* = 14) and EXP2 (*n* = 11), 5 patterns based on the seven virulence factors are shown in Table 2. The virulence factor *hasC* was present in all isolates in this study. From EXP1, four virulence factor patterns were found from 14 isolates, indicating variation of virulence patterns for *S. uberis*. In contrast to persistent IMI, the majority of patterns for transient *S. uberis* did not include *hasA* together with *hasB* or *hasAB* (63.6%), as shown in patterns I and II. Pattern III as *sua*+*hasA*+*hasB*+*hasC*+*gapC*+*pauA*+*cfu* was a pattern for persistent IMI but also found in transient IMI. From EXP2, a high similarity of the virulence pattern within a PFGE strain was found. Pattern III of *S. uberis* PFGE type A accounted for 81.8 including 60, and 100% for transient and persistent IMI, respectively. Few changes in virulence pattern (18.2%) to patterns IV (*n* = 1) and V (*n* = 1) were found and were related to their subsequently transient IMI appearance.

**Table 2:** Virulence patterns of *Streptococcus uberis* obtained from a weekly longitudinal study in experiment 1 (EXP1) and from a stored-known-appearance PFGE strain of a contagious *S. uberis* in experiment 2 (EXP2) between transient and persistent intramammary infection (IMI).

**IMI duration**	**%**	**VF pattern**	***sua***	***hasA***	***hasB***	***hasC***	***gapC***	***pauA***	***cfu***
**Experiment 1 (*****S. uberis*** **from a weekly longitudinal study)**									
Transience (*n* = 11)	9.1	I				^*^			
	54.5	II	^*^			^*^	^*^	^*^	^*^
	18.2	III	^*^	^*^	^*^	^*^	^*^	^*^	
	18.2	IV	^*^	^*^	^*^	^*^	^*^	^*^	^*^
Persistence (*n* = 3)	100	III	^*^	^*^	^*^	^*^	^*^	^*^	
**Experiment 2 (*****S. uberis*** **from a stored-known-appearance PFGE strain of a contagious** ***S. uberis*****)**									
Transience (*n* = 5)	60	III	^*^	^*^	^*^	^*^	^*^	^*^	
	20	IV	^*^	^*^	^*^	^*^	^*^	^*^	^*^
	20	V	^*^	^*^	^*^	^*^		^*^	
Persistence (*n* = 6)	100	III	^*^	^*^	^*^	^*^	^*^	^*^	

* *Positive PCR results*.

## Discussion

Because both farms used as sources of EXP1 and EXP2 were smallholder dairy farms with a high prevalence *S. uberis* mastitis, they might not be the representative farms in some circumstances. Therefore, the implications of our results must be carefully considered. From EXP1, spontaneous cure rates at 50 within 1 week and 75% within 10 weeks of *S. uberis* were higher and shorter than those at 50% within 10 months as reported in our previous study (22). However, both *S. uberis* IMI durations were in the range of IMI duration reported in many studies, for example, 50 to 260 days in a UK study (2), 1 to 309 days in a Dutch study (23), and 1 to 370 days in a US study (19). Within a week, 50% of spontaneous cures occurred, as in the transient cases in this study (Figure 1), which might indicate that most cases of *S. uberis* in this study were not contagious and likely arise from the cows' environment, as found in most studies (24–26).

Our previous molecular study showed that environmental *S. uberis* had a high rate and short duration of spontaneous cure (1). All 25 *S. uberis* isolates were confirmed by analysis of the 16S rRNA gene as a useful tool for more precise identification of streptococci in bovine milk (7). From EXP1, patterns I and II, the patterns without *hasAB* gene, the essential genes for capsule formation (27) were mostly found for transient *S. uberis* IMI. This finding on virulence patterns I and II in transient *S. uberis* was supported by Ward and Field (8), who demonstrated that the absence of the complete *hasA*+*hasB*+*hasC* or *hasAB*C or a capsule was considered to reduce virulence with a consequent reduction in resistance to the bactericidal action of neutrophils. The disruption of *hasA* or *hasC* gene resulted in the incomplete cessation of HA capsule development (8, 20). In this study, the virulence pattern with the presence of *cfu* gene was found only in transient *S. uberis* IMI. Previous studies have been reported that beta-hemolysin or cytolysins can promote invasion of host cells and also induce inflammatory responses and apoptosis by inducing the formation of neutrophil extracellular traps (NETs) (28, 29). Our finding was consistent with the study by Sagar et al. (30) who demonstrated that the non-hemolytic *Streptococcus* strains were able to survive in significantly higher numbers than the hemolytic strains and may contribute to the increased intracellular persistence. However, the study about hemolytic ability in the udder environment was very limited. Pattern III was the only virulence pattern found in the persistent *S. uberis* isolates. The major virulence patterns in EXP1 *S. uberis*, patterns III and IV, were also found to be the most common virulence patterns in Argentina (7). The virulence genes found in pattern III, *sua*+*hasABC*+*gapC*+*pauA*, encode virulence factors that promote essential mechanisms for survival. There was adhesion to/invasion of host tissue, evasion of the immune response, internalization in the mammary gland cells, and survival in the host environment, suggesting that *S. uberis* with this pattern may be more virulent and have a greater probability of contributing to chronic infection.

EXP2 was the first to determine virulence factor patterns between transient and persistent IMI of contagious *S. uberis* strains in the same PFGE type. The use of PFGE was verified to be a highly discriminatory method for detecting differences in strains of *S. uberis* (31). These *S. uberis* PFGE type A strains were categorized into a wide range of duration of infection where spontaneous cure was possible depending on host defense capacity (1). Most virulence patterns in this PFGE type were pattern III (81.8%) with some different gene profiles (18.2%; patterns IV and V), and the latter was found only in their transient IMI. Our finding was supported by the recent DNA microarray or PCR studies indicating similar virulence gene profiles in isolates with the same sequence type (32–34). These different gene profiles in the same genotype might relate to the reprogramming of the virulence genes by the udder environment (35). In addition, the different gene profiles in the same genotype might relate to the limitation of the discriminatory power of PFGE (36). However, pattern III appeared to be the majority in both transient (60%) and persistent IMI (100%), indicating that the udder immune response might play a role in their appearances (1).

## Conclusion

In conclusion, our study showed variability of virulence patterns for *S. uberis*. Based on samples from a dairy farm, transient IMI of *S. uberis* (63.6%) mostly had no capsule formation gene (*hasABC*). A representative of a contagious *S. uberis* PFGE strain showed a high similarity of virulence patterns with few changes relating to subsequent transient IMI. However, the majority of virulence patterns in this strain appeared in both transient and persistent IMI, indicating that the udder immune response might play a role in their appearances.

## Data Availability Statement

The original contributions presented in the study are included in the article/supplementary material, further inquiries can be directed to the corresponding author.

## Author Contributions

AS and WS contributed to conception and design of the study and wrote the first draft of the manuscript. AS and NP organized the database. WS revised the final draft of the manuscript. All authors contributed to manuscript revision, read, and approved the submitted version.

## Funding

This research was funded by the Royal Golden Jubilee (RGJ) Ph.D. Programme (Grant number: 5.VS.CM/61/D.1).

## Conflict of Interest

The authors declare that the research was conducted in the absence of any commercial or financial relationships that could be construed as a potential conflict of interest.

## Publisher's Note

All claims expressed in this article are solely those of the authors and do not necessarily represent those of their affiliated organizations, or those of the publisher, the editors and the reviewers. Any product that may be evaluated in this article, or claim that may be made by its manufacturer, is not guaranteed or endorsed by the publisher.

## References

[1] Leelahapongsathon K Schukken Y Srithanasuwan A Suriyasathaporn W. Molecular epidemiology of *Streptococcus uberis* intramamary infections: persistent and transient patterns of infection in a dairy herd. J Dairy Sci. (2020) 103: 3565–76. 10.3168/jds.2019-17281 32037160

[2] Pullinger G Coffey T Maiden M Leigh J. Multilocus-sequence typing analysis reveals similar populations of *Streptococcus uberis* are responsible for bovine intramammary infections of short and long duration. Vet Microbiol. (2007) 119:194–204. 10.1016/j.vetmic.2006.08.015 16973306

[3] Thompson-Crispi K Atalla H Miglior F Mallard BA. Bovine mastitis: frontiers in immunogenetics. Front Immunol. (2014) 5:493. 10.3389/fimmu.2014.00493 25339959 PMC4188034

[4] Zadoks R Gillespie B Barkema H Sampimon O Oliver S Schukken YJE. Clinical, epidemiological, and molecular characteristics of Streptococcus uberis infections in dairy herds. Epidemiol Infect. (2003) 130:335–49. 10.1017/s0950268802008221 12729202 PMC2869969

[5] Suriyasathaporn W Heuer C Noordhuizen-Stassen E Schukken Y. Hyperketonemia and the impairment of udder defense: A review. Vet Res. (2000) 31:397–412. 10.1051/vetres:2000128 10958241

[6] Burvenich C Van Merris V Mehrzad J Diez-Fraile A Duchateau LJ. Severity of *E. coli* mastitis is mainly determined by cow factors. Vet Res. (2003) 34:521–64. 10.1051/vetres:2003023 14556694

[7] Reinoso EB Lasagno MC Dieser SA Odierno LM. Distribution of virulence-associated genes in *Streptococcus uberis* isolated from bovine mastitis. FEMS Microbiol Lett. (2011) 318:183–8. 10.1111/j.1574-6968.2011.02258.x 21385204

[8] Ward PN Field TR Ditcham WG Maguin E Leigh JA. Identification and disruption of two discrete loci encoding hyaluronic acid capsule biosynthesis genes *hasA, hasB*, and *hasC* in *Streptococcus uberis*. Infect Immun. (2001) 69:392–9. 10.1128/IAI.69.1.392-399.2001 11119529 PMC97895

[9] Rosey EL Lincoln RA Ward PN Yancey RJ Jr Leigh JA. PauA: a novel plasminogen activator from Streptococcus uberis FEMS. Microbiol Lett. (1999) 178:27–33. 10.1111/j.1574-6968.1999.tb13755.x %J FEMS Microbiology Letters 10483719

[10] Almeida RA Oliver SP. Trafficking of Streptococcus uberis in bovine mammary epithelial cells. Microb Pathog. (2006) 41:80–9. 10.1016/j.micpath.2006.04.007 16872802

[11] Pancholi V Fischetti VA. A major surface protein on group A streptococci is a glyceraldehyde-3-phosphate-dehydrogenase with multiple binding activity. J Exp Med. (1992) 176:415–26. 10.1084/jem.176.2.415 1500854 PMC2119316

[12] Chen X Dego OK Almeida RA Fuller TE Luther DA Oliver SP. Deletion of sua gene reduces the ability of Streptococcus uberis to adhere to and internalize into bovine mammary epithelial cells. Vet Microbiol. (2011) 147:426–34. 10.1016/j.vetmic.2010.07.006 20708860

[13] Leigh JA Lincoln RA. *Streptococcus uberis* acquires plasmin activity following growth in the presence of bovine plasminogen through the action of its specific plasminogen activator. FEMS Microbiol Lett. (1997) 154:123–9. 10.1111/j.1574-6968.1997.tb12633.x 9297830

[14] Leigh J Egan S Ward P Field T Coffey T. Sortase anchored proteins of *Streptococcus uberis* play major roles in the pathogenesis of bovine mastitis in dairy cattle. Vet Res. (2010) 41:63. 10.1051/vetres/2010036 20519112 PMC2898060

[15] Loures R Pereira U Carvalho-Castro G Mian G Custódio D Silva J. Genetic diversity and virulence genes in *Streptococcus uberis* strains isolated from bovine mastitis. Semina: Ciências Agrárias. (2017) 38:2595. 10.5433/1679-0359.2017v38n4SUPLp2595

[16] Moliva MV Campra N Ibañez M Cristofolini AL Merkis CI Reinoso EB. Capacity of adherence, invasion and intracellular survival of *Streptococcus uberis* biofilm-forming strains. J Appl Microbiol. (2121) 132:1–9. 10.1111/jam.15362 34800320

[17] Constable PD Hinchcliff KW Done SH Grünberg W. Veterinary medicine-e-book: a textbook of the diseases of cattle, horses, sheep, pigs and goats. Elsevier Health Sciences. (2016).

[18] Goel MK Khanna P Kishore J. Understanding survival analysis: Kaplan-Meier estimate. Int J Ayurveda Res. (2010) 1:274–8. 10.4103/0974-7788.76794 21455458 PMC3059453

[19] Shome BR Das Mitra S Bhuvana M Krithiga N Velu D Shome R. Multiplex PCR assay for species identification of bovine mastitis pathogens. Appl Microbiol. (2011) 111:1349–56. 10.1111/j.1365-2672.2011.05169.x 21972842

[20] Field TR Ward PN Pedersen LH Leigh JA. The hyaluronic acid capsule of *Streptococcus uberis* is not required for the development of infection and clinical mastitis. Infect Immun. (2003) 71:132–9. 10.1128/iai.71.1.132-139.2003 12496158 PMC143150

[21] Shome B Bhuvana M Das Mitra S Krithiga N Shome R Velu D. Molecular characterization of Streptococcus agalactiae and Streptococcus uberis isolates from bovine milk. Trop Anim Health Prod. (2012) 44:1981–92. 10.1007/s11250-012-0167-4 22588571

[22] Leelahapongsathon K Schukken YH Pinyopummintr T Suriyasathaporn W. Comparison of transmission dynamics between Streptococcus uberis and Streptococcus agalactiae intramammary infections. J Dairy Sci. (2016) 99:1418–26. 10.3168/jds.2015-9950 26686709

[23] Todhunter DA Smith KL Hogan JS. Environmental streptococcal intramammary infections of the bovine mammary gland1. J Dairy Sci. (1995) 78:2366–74. 10.3168/jds.S0022-0302(95)76864-3 8747327

[24] Khan IU Hassan AA Abdulmawjood A Lämmler C Wolter W Zschöck M. Identification and epidemiological characterization of *Streptococcus uberis* isolated from bovine mastitis using conventional and molecular methods. J Vet Sci. (2003) 4:213–24. 10.4142/jvs.2003.4.3.213 14685025

[25] McDougall S Parkinson TJ Leyland M Anniss FM Fenwick SG. Duration of infection and strain variation in *Streptococcus uberis* isolated from cows' milk. J Dairy Sci. (2004) 87:2062–72. 10.3168/jds.S0022-0302(04)70024-7 15328218

[26] Abureema S Smooker P Malmo J Deighton M. Molecular epidemiology of recurrent clinical mastitis due to *Streptococcus uberis*: evidence of both an environmental source and recurring infection with the same strain. J Dairy Sci. (2014) 97:285–90. 10.3168/jds.2013-7074 24239086

[27] Coffey TJ Pullinger GD Urwin R Jolley KA Wilson SM Maiden MC. First insights into the evolution of *Streptococcus uberis*: a multilocus sequence typing scheme that enables investigation of its population biology. Appl Environ Microbiol. (2006) 72:1420–8. 10.1128/AEM.72.2.1420-1428.2006 16461695 PMC1392973

[28] Zhong H Yazdanbakhsh K. Hemolysis and immune regulation. Curr Opin Hematol. (2018) 25:177–82. 10.1097/MOH.0000000000000423 29461260 PMC6309361

[29] Kabelitz T Aubry E van Vorst K Amon T Fulde M. The role of *Streptococcus* spp. in Bovine Mastitis. (2021) 9:1497. 10.3390/microorganisms9071497 34361932 PMC8305581

[30] Sagar A Klemm C Hartjes L Mauerer S van Zandbergen G Spellerberg B. The β-Hemolysin and intracellular survival of *Streptococcus agalactiae* in human macrophages. PLoS ONE. (2013) 8:e60160. 10.1371/journal.pone.0060160 23593170 PMC3617175

[31] Gillespie B Oliver S. Comparison of an automated ribotyping system, pulsed-field gel electrophoresis and randomly amplified polymorphic DNA fingerprinting for differentiation of *Streptococcus uberis* strains. Biotechnology. (2004) 3:165–72.

[32] Liew YK Hamat R Amin Nordin S Chong P Neela V. The exoproteomes of clonally related *Staphylococcus aureus* strains are diverse. Ann Microbiol. (2015) 65. 10.1007/s13213-015-1064-7

[33] Shambat S Nadig S Prabhakara S Bes M Etienne J Arakere GJ. Clonal complexes and virulence factors of *Staphylococcus aureus* from several cities in India. BMC Microbiol. (2012) 12:64. 10.1186/1471-2180-12-64 22548694 PMC3438138

[34] Jamrozy DM Fielder MD Butaye P Coldham NG. Comparative genotypic and phenotypic characterisation of methicillin-resistant staphylococcus aureus ST398 isolated from animals and humans. PLoS ONE. (2012) 7:e40458. 10.1371/journal.pone.0040458 22792335 PMC3394705

[35] Thomas MS Wigneshweraraj S. Regulation of virulence gene expression. Virulence. (2014) 5:832–4. 10.1080/21505594.2014.995573 25603428 PMC4601333

[36] Dudek B Ksiazczyk M Krzyzewska E Rogala K Kuczkowski M Wozniak-Biel A. Comparison of the phylogenetic analysis of PFGE profiles and the characteristic of virulence genes in clinical and reptile associated Salmonella strains. BMC Vet Res. (2019) 15:312. 10.1186/s12917-019-2019-1 31477105 PMC6721270

